# Italian legumes: effect of sourdough fermentation on lunasin-like polypeptides

**DOI:** 10.1186/s12934-015-0358-6

**Published:** 2015-10-22

**Authors:** Carlo Giuseppe Rizzello, Blanca Hernández-Ledesma, Samuel Fernández-Tomé, José Antonio Curiel, Daniela Pinto, Barbara Marzani, Rossana Coda, Marco Gobbetti

**Affiliations:** Department of Soil, Plant and Food Science, University of Bari Aldo Moro, 70126 Bari, Italy; Instituto de Investigación en Ciencias de la Alimentación (CIAL, CSIC-UAM CEI UAM + CSIC), Nicolás Cabrera, 9, 28049 Madrid, Spain; Giuliani S.p.a., Milano, Italy; Department of Food and Environmental Sciences, University of Helsinki, Helsinki, Finland

**Keywords:** Legumes, Sourdough, Lactic acid bacteria, Lunasin

## Abstract

**Background:**

There is an increasing interest toward the use of legumes in food industry, mainly due to the quality of their protein fraction. Many legumes are cultivated and consumed around the world, but few data is available regarding the chemical or technological characteristics, and especially on their suitability to be fermented. Nevertheless, sourdough fermentation with selected lactic acid bacteria has been recognized as the most efficient tool to improve some nutritional and functional properties. This study investigated the presence of lunasin-like polypeptides in nineteen traditional Italian legumes, exploiting the potential of the fermentation with selected lactic acid bacteria to increase the native concentration. An integrated approach based on chemical, immunological and ex vivo (human adenocarcinoma Caco-2 cell cultures) analyses was used to show the physiological potential of the lunasin-like polypeptides.

**Results:**

Italian legume varieties, belonging to *Phaseulus vulgaris*, *Cicer arietinum*, *Lathyrus sativus*, *Lens culinaris* and *Pisum sativum* species, were milled and flours were chemically characterized and subjected to sourdough fermentation with selected *Lactobacillus plantarum* C48 and *Lactobacillus brevis* AM7, expressing different peptidase activities. Extracts from legume doughs (unfermented) and sourdoughs were subjected to western blot analysis, using an anti-lunasin primary antibody. Despite the absence of lunasin, different immunoreactive polypeptide bands were found. The number and the intensity of lunasin-like polypeptides increased during sourdough fermentation, as the consequence of the proteolysis of the native proteins carried out by the selected lactic acid bacteria. A marked inhibitory effect on the proliferation of human adenocarcinoma Caco-2 cells was observed using extracts from legume sourdoughs. In particular, sourdoughs from Fagiolo di Lamon, Cece dell’Alta Valle di Misa, and Pisello riccio di Sannicola flours were the most active, showing a decrease of Caco-2 cells viability up to 70 %. The over-expression of Caco-2 filaggrin and involucrin genes was also induced. Nine lunasin-like polypeptides, having similarity to lunasin, were identified.

**Conclusions:**

The features of the sourdough fermented legume flours suggested the use for the manufacture of novel functional foods and/or pharmaceuticals preparations.

## Background

According to the Food and Agricultural Organization (FAO) pulses are dry seeds of annual legume plants, belonging to the *Fabaceae* (also known as *Leguminosae*) family. FAO classifies the large number of legume species and varieties employed as food or feed into eleven main groups (dry beans, dry broad beans, dry peas, chickpeas, dry cowpeas, pigeon peas, lentils, bambara beans, vetches and lupins) and minor pulses [1]. Nutritionally, pulses are an important source of proteins, which, in spite of being deficient in sulfur-containing amino acids and tryptophan, possess higher amounts of lysine, arginine, glutamic, and aspartic acid compared to cereal grains [2]. Beyond the nutritional benefits, consumption of pulses is recently associated with protective or therapeutic effects on chronic health conditions, such as cardiovascular diseases, diabetes, cancer, overweight, and obesity [2]. Owing to the low cost and easy adaptation to grow under poor conditions, pulses are used as staple foods in several low-income countries, serving as main source of both protein and calories [2, 3]. Otherwise, in high-income countries of America and Europe, pulses consumption is low and efforts are done to promote their healthy intake.

Several health organizations recommend pulse consumption as a part of a healthy diet and initiatives are addressed to increase the cultivation, intake, and food processing uses [2, 4]. The protein content of pulses ranges from 20 to 40 % of dry weight. Within this interval, the most abundant are the seed storage proteins. The remaining part are minor or housekeeping proteins, which include enzymes, protease, amylase inhibitors, lectins, lipoxygenases, defense proteins, and others [3, 5]. Legume seeds also contain proteins, which are considered as anti-nutritional compounds due to the effect on the quality of the diet. Nevertheless, the harmful effects of such compounds is easily inactivated after cooking or processes like fermentation, germination and dehulling [6]. Once inactivated, lectins or protease inhibitors may present potential health benefits. Protease inhibitors are potential anti-inflammatory and anticancer agents, whereas lectins have demonstrated to play a key role in preventing certain cancers and activating innate defense mechanisms. Besides, lectins are also proposed as therapeutic agents to prevent or control obesity [5].

Many of the physiological and functional properties of proteins are attributed to biologically active peptides which are often encrypted in the native sequence [7, 8]. Biogenic or bioactive peptides are released from their precursor proteins either by enzymes during gastrointestinal digestion or through proteolysis (e.g., microbial fermentation), which occurs during food processing [8]. In particular, legume hydrolysates and bioactive peptides had in vitro activities towards cancer and cardiovascular diseases or their physiological manifestations like oxidative damage, inflammation, hypertension, and high cholesterol [2]. Lunasin is a 43-amino acid peptide with anticancer, anti-inflammatory, antioxidant and cholesterol lowering activities [9]. It is purified from soybean and commercialized as an ingredient or dietary supplement. In the quest for readily available natural sources of lunasin, the identification and purification of lunasin from different vegetable sources deserve a marked interest [10, 11]. Moreover, the potential of sourdough fermentation for increasing the concentration of lunasin in food matrices was recently investigated [12]. Sourdough is the natural starter traditionally used for making leavened baked goods, harboring a rich lactic acid bacteria and yeast microbiota. A large number of studies [13] showed that the fermentative and proteolytic activities of sourdough lactic acid bacteria not only determined optimal sensory, technology and nutritional characteristics, but also increased the functional value of leavened baked goods. Compared to unfermented soybean, amaranth, barley and wheat flours, the concentration of lunasin and related fragments increased up to four times during fermentation with lactic acid bacteria, which were selected based on proteolytic activities [12].

This study reported the presence of lunasin-like polypeptides in nineteen traditional Italian legumes, and exploited the potential of the fermentation with selected lactic acid bacteria to increase the native concentration. An integrated approach based on chemical, immunological and ex vivo (human adenocarcinoma Caco-2 cell cultures) analyses was used to show the physiological potential of the lunasin-like polypeptides.

## Results

### Chemical and microbiological characteristics of the flours

All the traditional Italian legumes used in this study have specific certifications (names, abbreviations, geographical origin and certification are listed in Table 1). Legume grains were milled to obtain the corresponding flours. The proximate composition of the flours is reported in Table 2. Moisture ranged from 7.2 ± 0.4 to 11.1 ± 0.7 %. The protein concentration of all the flours was higher than 15 % of dry matter (d.m.). In particular, grass pea flours (CS and CC) showed the highest values (>24.0 %). The lipid concentration varied from 1.4 ± 0.2 to 3.5 ± 0.1 % of d.m., with the exception of chickpea flours (CM and CV), showing a significantly (*P* < 0.05) higher concentration. All flours had an amount of carbohydrates higher than 60 % of d.m. The highest concentration was found for FCo and LA flours, produced by milling Fagiolo di Controne kidney beans and Lenticchia di Altamura lentils, respectively. Legume flours had concentrations of total dietary fiber higher than 17 % of d.m. PS and CC flours had the highest values (35.5 ± 2.9 and 32.1 ± 2.0 %, respectively), whereas the lentils group contained the lowest value of total dietary fiber. Ash ranged from 2.1 ± 0.2 to 4.6 ± 0.3 %, being the lowest values for lentil flours.

**Table 1:** List and abbreviations of the Italian legumes

Legume	Name	Abbreviation	Product certification^a^	Origin
Kidney bean (*Phaseolus vulgaris*)	Fagiolo di Lamon	FL	IGP	Veneto
	Fagiolo di Controne	FCo	DOP	Campania
	Fagiolo di Cuneo	FCu	PAT	Piedmont
	Fagiolo Stregoni	FSt	PAT	Piedmont
	Fagiolo Vellutina	FV	PAT	Sicily
	Fagiolo di Saluggia	FSa	PAT	Piedmont
	Fagiolo Badda di Polizzi (white)	FBw	SFP	Sicily
	Fagiolo Badda di Polizzi (black)	FBb	SFP	Sicily
Chickpea (*Cicer arietinum*)	Cece di Merella	CM	PAT	Piedmont
	Cece dell’Alta Valle del Misa	CV	SFP	Marche
Grass pea (*Lathyrus sativus*)	Cicerchia di Serra de Conti	CS	SFP	Marche
	Cicerchia di Campodimele	CC	PAT	Lazio
Lentil (*Lens culinaris*)	Lenticchia di Castelluccio di Norcia	LN	IGP	Umbria
	Lenticchia di Ustica	LU	SFP	Sicily
	Lenticchia di Santo Stefano di Sessanio	LS	SFP	Abruzzo
	Lenticchia rossa di Pantelleria	LP	PAT	Sicily
	Lenticchia di Altamura	LA	PAT	Apulia
	Lenticchia di Villalba	LV	PAT	Sicily
Pea (*Pisum sativum*)	Pisello riccio di Sannicola	PS	PAT	Apulia

Product certifications and origin are also reported

^a^IGP (Indicazione Geografica Protetta, Protected Geographical Indication) and DOP (Denominazione d’Origine Protetta, Designation of Protected Origin) are regulated by Reg. (CE) N. 510/2006 (20.03.2006); PAT (Prodotti Agroalimentari Tradizionali, Traditional Food Products) are included in the list of the Italian Ministry of Agriculture, Food and Forestry (D.M. 07/06/2012); SFP (Slow Food Presidia) are listed at http://www.slowfoodfoundation.org

**Table 2:** Proximate composition of the Italian legume flours

		Moisture (%)	Proteins (% of d.m.)	Lipids (% of d.m.)	Carbohydrates (% of d.m.)	Dietary fiber (% of d.m.)	Ash (% of d.m.)
FL	Fagiolo di Lamon	7.2 ± 0.4^e^	20.2 ± 1.5^b^	3.4 ± 0.2^b^	65.0 ± 3.5^c^	21.2 ± 1.5^d^	4.0 ± 0.4^b^
FCo	Fagiolo di Controne	7.2 ± 0.5^e^	17.3 ± 1.5^d^	2.7 ± 0.3^c^	69.2 ± 5.0^a^	26.0 ± 1.3^b^	3.5 ± 0.2^c^
FCu	Fagiolo di Cuneo	8.0 ± 0.8^d^	20.5 ± 2.0^b^	2.0 ± 0.4^d^	65.2 ± 3.0^c^	24.3 ± 2.3^c^	3.5 ± 0.3^b^
FSt	Fagiolo Stregoni	10.2 ± 0.7^b^	17.2 ± 1.5^e^	2.3 ± 0.2^c^	67.3 ± 2.5^b^	22.6 ± 2.2^c^	3.3 ± 0.2^c^
FV	Fagiolo Vellutina	11.1 ± 0.7^a^	18.6 ± 1.4^d^	2.5 ± 0.3^c^	64.5 ± 5.0^c^	26.5 ± 1.9^b^	3.8 ± 0.1^b^
FSa	Fagiolo di Saluggia	10.9 ± 0.8^a^	18.4 ± 0.6^d^	2.6 ± 0.4^c^	65.8 ± 4.0^c^	22.7 ± 1.7^c^	3.0 ± 0.1^c^
FBw	Fagiolo Badda di Polizzi (white)	7.3 ± 0.2^e^	21.0 ± 1.4^b^	2.6 ± 0.3^c^	65.4 ± 3.6^c^	19.6 ± 2.3^d^	3.6 ± 0.2^b^
FBb	Fagiolo Badda di Polizzi (black)	8.5 ± 0.5^d^	19.0 ± 1.8^c^	3.5 ± 0.1^b^	65.2 ± 3.4^c^	19.4 ± 0.9^d^	3.6 ± 0.2^b^
CM	Cece di Merella	8.8 ± 0.5^d^	15.7 ± 1.0^e^	6.2 ± 0.4^a^	66.5 ± 3.6^b^	27.5 ± 1.1^b^	3.5 ± 0.3^c^
CV	Cece Alta Valle di Misa	8.9 ± 0.3^d^	20.0 ± 0.8^c^	6.3 ± 0.1^a^	61.2 ± 3.5^e^	26.8 ± 2.3^b^	4.0 ± 0.2^b^
CS	Cicerchia di Serra de Conti	9.2 ± 0.5^c^	24.3 ± 1.5^a^	1.4 ± 0.2^e^	60.8 ± 3.5^e^	25.1 ± 1.2^c^	3.7 ± 0.4^b^
CC	Cicerchia di Campodimele	9.3 ± 0.6^c^	24.1 ± 2.0^a^	2.2 ± 0.4^d^	61.5 ± 3.1^e^	32.1 ± 2.0^a^	3.8 ± 0.2^b^
LN	Lenticchia di Castelluccio di Norcia	8.2 ± 0.7^d^	23.5 ± 1.2^a^	3.0 ± 0.5^b^	62.8 ± 2.5^d^	18.2 ± 1.9^e^	2.6 ± 0.1^d^
LU	Lenticchia di Ustica	8.8 ± 0.4^d^	23.0 ± 1.0^a^	3.4 ± 0.2^b^	62.6 ± 3.7^d^	17.6 ± 2.1^e^	2.3 ± 0.2^e^
LS	Lenticchia di Santo Stefano di Sessanio	8.7 ± 0.8^d^	22.0 ± 1.3^b^	3.2 ± 0.2^b^	64.1 ± 3.0^d^	25.2 ± 1.9^c^	3.0 ± 0.3^d^
LP	Lenticchia rossa di Pantelleria	8.0 ± 0.5^d^	21.2 ± 1.5^b^	2.7 ± 0.1^c^	65.9 ± 2.9^c^	20.5 ± 1.1^d^	2.2 ± 0.1^e^
LA	Lenticchia di Altamura	7.4 ± 0.2^e^	18.7 ± 0.9^d^	2.4 ± 0.2^d^	69.1 ± 2.2^a^	22.8 ± 1.8^c^	2.5 ± 0.2^d^
LV	Lenticchia di Villalba	8.6 ± 0.5^d^	20.8 ± 1.5^b^	2.1 ± 0.2^d^	65.9 ± 3.0^b^	17.5 ± 0.4^e^	2.1 ± 0.2^e^
PS	Pisello riccio di Sannicola	9.2 ± 0.7^c^	19.6 ± 1.0^c^	2.2 ± 0.2^d^	65.3 ± 2.0^c^	35.5 ± 2.9^a^	4.6 ± 0.3^a^

The data are the means of three independent experiments ± standard deviations (n = 3)

*d.m.* dry matter

^a–e^Values in the same column with different superscript letters differ significantly (*P* < 0.05)

Total mesophilic aerobic bacteria ranged from 1.61 ± 0.32 to 4.31 ± 0.11 log cfu/g (Table 3). Enterobacteria were below 2.0 log cfu/g in all the samples. The number of presumptive lactic acid bacteria varied from 1.0 ± 0.08 to 2.54 ± 0.12 log cfu/g. Except for grass pea flour CC (4.23 ± 0.22 log cfu/g), yeasts were 1.11 ± 0.05–2.77 ± 0.20 log cfu/g. Yeasts were not found in 10 g of LP, FV, FSa, and FBb flours. Molds were found at 2.0 ± 0.21–3.51 ± 0.13 log cfu/g in most of the flours and were absent in 10 g of LS, FCo, CC, and PS flours.

**Table 3:** Microbiological analyses of the Italian legume flours

Legume	Total mesophilic aerobic bacteria	Lactic acid bacteria	Yeasts	Molds	Total enterobacteria
FL	3.52 ± 0.12^b^	1.93 ± 0.12^b^	2.77 ± 0.20^c^	3.51 ± 0.13ª	0.82 ± 0.11^b^
FCo	3.60 ± 0.20^b^	2.49 ± 0.22ª	1.84 ± 0.20^d^	nf	0.53 ± 0.09^c^
FCu	3.23 ± 0.22^b^	2.54 ± 0.12ª	1.11 ± 0.05^e^	3.33 ± 0.21ª	0.82 ± 0.11^b^
FSt	4.21 ± 0.18ª	2.25 ± 0.23ª	2.14 ± 0.21^c^	3.31 ± 0.22ª	0.91 ± 0.07^b^
FV	2.92 ± 0.13^c^	1.14 ± 0.24^c^	nf	2.81 ± 0.15^b^	0.51 ± 0.09^c^
FSa	2.55 ± 0.25^c^	1.16 ± 0.11^c^	nf	2.47 ± 0.15^c^	0.62 ± 0.13^c^
FBw	3.34 ± 0.19^b^	1.47 ± 0.15^c^	1.30 ± 0.21^e^	3.23 ± 0.16^a^	0.73 ± 0.09^b^
FBb	1.61 ± 0.32^d^	1.00 ± 0.16^c^	nf	2.45 ± 0.15^c^	0.82 ± 0.09^b^
CM	2.84 ± 0.22^c^	1.31 ± 0.18^c^	1.69 ± 0.23^d^	2.47 ± 0.22^c^	0.91 ± 0.14^b^
CV	2.47 ± 0.14^c^	1.47 ± 0.21^c^	1.95 ± 0.09^c^	2.30 ± 0.24^c^	0.9 ± 0.09^b^
CS	2.25 ± 0.15^c^	1.69 ± 0.23^b^	nf	2.00 ± 0.21^c^	0.8 ± 0.10^b^
CC	2.32 ± 0.23^c^	1.84 ± 0.22^b^	4.23 ± 0.22^a^	nf	0.53 ± 0.05^c^
LN	2.69 ± 0.21^c^	1.77 ± 0.24^b^	1.47 ± 0.13^e^	2.04 ± 0.23^c^	1.32 ± 0.10^a^
LU	2.36 ± 0.24^c^	1.84 ± 0.25^b^	2.08 ± 0.15^c^	2.30 ± 0.20^c^	0.64 ± 0.11^c^
LS	2.95 ± 0.09^c^	1.47 ± 0.24^c^	2.11 ± 0.15^c^	nf	0.75 ± 0.13^b^
LP	4.31 ± 0.11^a^	1.30 ± 0.25^c^	nf	3.23 ± 0.20^a^	0.52 ± 0.22^c^
LA	3.69 ± 0.16^b^	1.30 ± 0.08^c^	3.50 ± 0.15^b^	2.11 ± 0.20^c^	0.72 ± 0.14^b^
LV	2.61 ± 0.17^c^	1.00 ± 0.08^c^	2.47 ± 0.12^c^	2.23 ± 0.15^c^	0.82 ± 0.12^b^
PS	2.47 ± 0.23^c^	1.30 ± 0.10^c^	1.30 ± 0.21^e^	nf	0.92 ± 0.13^b^

Total mesophilic aerobic bacteria were estimated on Plate Count Agar (PCA), lactic acid bacteria on agar MRS; yeasts and molds on Yeast extract Peptone Dextrose Agar (YPD-y and YPD-m, respectively); and total enterobacteria on Violet Red Bile Glucose Agar (VRBGA). Details are reported in “Methods” section

The data are the means of three independent experiments ± standard deviations (n = 3)

^a–e^Values in the same column with different superscript letters differ significantly (*P* < 0.05)

### Lactic acid bacteria fermentation

After incubation for 24 h at 30 °C, control doughs (D), without bacterial inoculum, showed values of pH that ranged from 5.8 ± 0.2 to 6.5 ± 0.3, corresponding to values of TTA of 3.8 ± 0.2 to 8.5 ± 0.5 ml 0.1 M NaOH/10 g of dough (Table 4). When *L. plantarum* C48 and *L. brevis* AM7 were used as starters for sourdough (S) fermentation, the cell density of presumptive lactic acid bacteria was 9.8–10.2 log cfu/g. All S had values of pH significantly (*P* < 0.05) lower (3.9 ± 0.1–4.5 ± 0.3) than the corresponding D. TTA ranged from 20.2 ± 1.3 to 27.2 ± 1.5 ml NaOH/10 g of dough.

**Table 4:** Biochemical characteristics of doughs

Legume	pH		TTA (ml of 0.1 M NAOH)		Free amino acids (mg/kg)	
	D	S	D	S	D	S
FL	6.5 ± 0.2^a^	4.4 ± 0.1^a^	5.7 ± 0.5^c^	26.6 ± 1.3^a^	4272 ± 10ª	5900 ± 18^b^
FCo	6.2 ± 0.1^b^	4.2 ± 0.2^c^	6.3 ± 0.2^b^	23.0 ± 0.9^c^	3348 ± 14c	5414 ± 17^c^
FCu	6.5 ± 0.3^a^	4.4 ± 0.2^a^	5.5 ± 0.3^c^	26.8 ± 2.1^a^	4018 ± 9ª	5605 ± 15^b^
FSt	6.4 ± 0.5^a^	4.5 ± 0.3^a^	3.1 ± 0.4^e^	22.7 ± 0.9^d^	3025 ± 11^c^	3982 ± 16^e^
FV	6.5 ± 0.2^a^	4.2 ± 0.2^c^	4.7 ± 0.3^d^	23.5 ± 1.8^c^	3991 ± 13^b^	5904 ± 12^b^
FSa	6.2 ± 0.2^b^	4.3 ± 0.1^b^	5.2 ± 0.4^c^	20.2 ± 1.3^e^	3512 ± 12^b^	3634 ± 18^e^
FBw	6.4 ± 0.3^a^	4.2 ± 0.1^c^	6.1 ± 0.6^b^	25.5 ± 1.8^b^	4547 ± 18ª	6620 ± 12ª
FBb	6.2 ± 0.6^b^	4.4 ± 0.2^a^	7.1 ± 0.5^a^	25.4 ± 1.5^b^	3901 ± 15^b^	6269 ± 14ª
CM	6.0 ± 0.1^c^	3.9 ± 0.1^e^	3.8 ± 0.2^e^	22.5 ± 1.0^d^	2550 ± 9^d^	2573 ± 16^f^
CV	6.2 ± 0.3^b^	4.1 ± 0.4^d^	5.1 ± 0.5^c^	22.3 ± 0.7^d^	2961 ± 13^c^	4524 ± 20^d^
CS	6.3 ± 0.2^b^	4.2 ± 0.1^c^	6.2 ± 0.3^b^	23.2 ± 1.2^c^	2652 ± 11^d^	4183 ± 17^d^
CC	6.0 ± 0.5^c^	4.2 ± 0.1^c^	7.4 ± 0.3^a^	27.2 ± 1.5^a^	2611 ± 13^d^	2730 ± 15^f^
LN	6.3 ± 0.5^b^	4.2 ± 0.2^c^	4.6 ± 0.4^d^	23.1 ± 1.4^c^	2825 ± 18^d^	3634 ± 18^e^
LU	6.0 ± 0.3^c^	4.0 ± 0.1^d^	4.2 ± 0.6^d^	22.2 ± 1.5^d^	2429 ± 20^d^	3274 ± 16^e^
LS	5.8 ± 0.2^d^	4.0 ± 0.1^d^	8.5 ± 0.2^a^	22.8 ± 2.0^d^	4324 ± 11ª	4743 ± 21^d^
LP	6.3 ± 0.4^b^	4.1 ± 0.4^d^	4.1 ± 0.2^d^	23.1 ± 1.5^c^	2631 ± 9^d^	3173 ± 11^e^
LA	6.3 ± 0.2^b^	4.1 ± 0.2^d^	3.9 ± 0.3^e^	22.5 ± 0.9^d^	3207 ± 13^c^	5104 ± 17^c^
LV	6.0 ± 0.2^c^	4.1 ± 0.3^d^	5.3 ± 0.2^c^	22.2 ± 1.1^d^	3196 ± 15^c^	5173 ± 22^c^
PS	6.3 ± 0.5^b^	4.4 ± 0.5^a^	4.3 ± 0.4^d^	20.4 ± 1.5^e^	3629 ± 16^b^	3686 ± 19^e^

Control doughs (D), without bacterial inoculation, and sourdoughs (S), started with selected lactic acid bacteria, made with the Italian legume flours were incubated at 30 °C for 24 h

The data are the means of three independent experiments ± standard deviations (n = 3)

^a–f^Values in the same column with different superscript letters differ significantly (*P* < 0.05)

The concentration of total free amino acids (FAA) of D and S is reported in Table 4. Before incubation, doughs had concentrations of total FAA varying from 2429 ± 20 (D made with LU flour) to 4547 ± 18 mg/kg (D made with FBw flour). In several cases, the concentration of total free amino acids (FAA) of S was significantly (*P* < 0.05) higher than that of the corresponding D. The average increase was ca. 28 % for kidney bean and pea S. Almost the same average increases (23–26 %) were found for grass pea, chickpea, and lentil S. Compared to D, slight increases of total FAA were shown by FSa, CM, CC, LS, and PS sourdoughs.

### Western blot

**Fig. 1:**
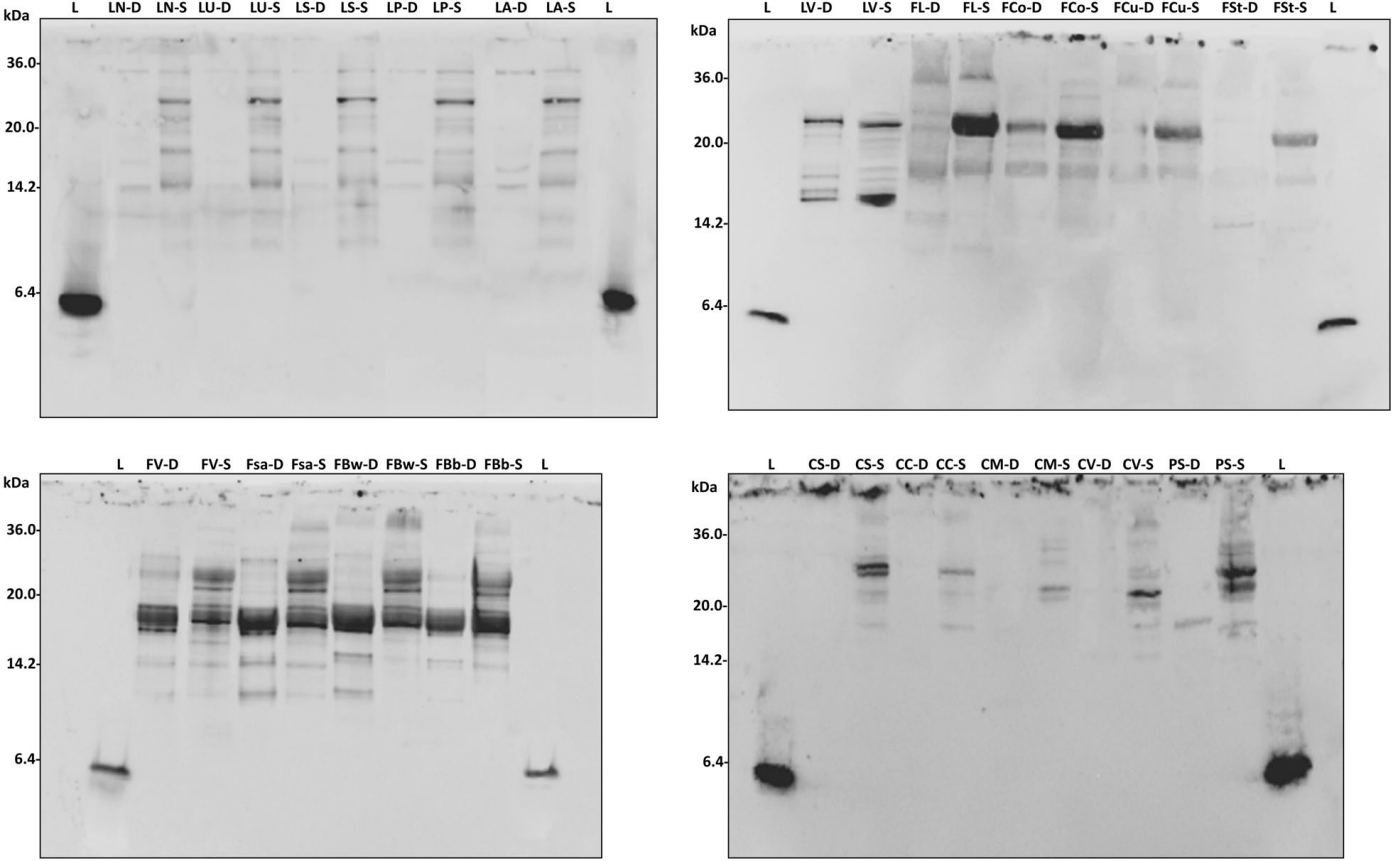
Western blot analysis. Water/salt soluble extracts obtained from control doughs (D), without bacterial inoculum, and sourdoughs (S), started with selected lactic acid bacteria, made with Italian legume flours, were used. A lunasin polyclonal primary antibody was used. Before electroblotting, the electrophoretic separation was obtained by Tris-Tricine SDS-PAGE. Image was performed using the VersaDoc Imaging System (Bio-Rad). Synthetic lunasin (L) was included in the analysis. The correspondence of the legume flour abbreviations is reported in Table 1

The water/salt soluble extracts (WSE) of D and S were analyzed by SDS PAGE, and the proteins were electroblotted and detected after incubation with lunasin polyclonal primary antibody (Fig. 1). A polyclonal primary antibody was already used for identification and quantification of lunasin and its use proposed for optimized methods of detection [14]. Lunasin peptide was not found in any of the samples analyzed. Nevertheless, different immunoreactive bands, having molecular masses higher than lunasin, were found. Among D, grass pea and chickpea doughs did not shown any immunoreactive bands, while pea and all the lentil doughs (with the exception of LV) showed very weak signals, mainly distributed below 15 kDa. A large variability was found among the doughs made with bean flours. Very weak bands were found for Fl, FCo, FCu, and Fst, while a large protein band (molecular mass of ca. 17 kDa) was found for LV, FV, FSa, FBw, and FBb.

Lactic acid bacteria fermentation induced a large modification of the profiles of immunoreactive protein of all the legume flours. A band of ca. 30 kDa, which was absent in the corresponding D, was found for LN, LU, LS, LP, and LA sourdoughs. The intensity of the 17 kDa band of fermented LV increased compared to D. The same signal became evident in all bean sourdoughs, especially for FL. Two protein bands were detected at ca. 30 kDa in FV, FSa, FBw and FBb. The same was found for CS and PS. Moreover, FV, FSa, FBw and FBb showed reactive bands also having molecular masses of 14–17 kDa.

### Effect on proliferation of Caco-2 cells

Aiming at determining the cytotoxicity effect of the freeze-dried WSE from D and S towards human colon adenocarcinoma cells (Caco-2), five samples (FL, CV, CC, LN, and PS) with different immunoreactive protein profiles, were chosen as representatives of the legume species considered in this study, and were subjected to further characterization. On the basis of the results obtained from the western blot analysis, WSE were partially purified by ultra-filtration, collecting the fractions containing the molecules with molecular mass lower than 30 kDa. In particular, the MTT assay was performed after treatment of Caco-2 cells with 0.1, 1, and 10 mg/ml of proteins for 24, 48, or 72 h.

**Fig. 2:**
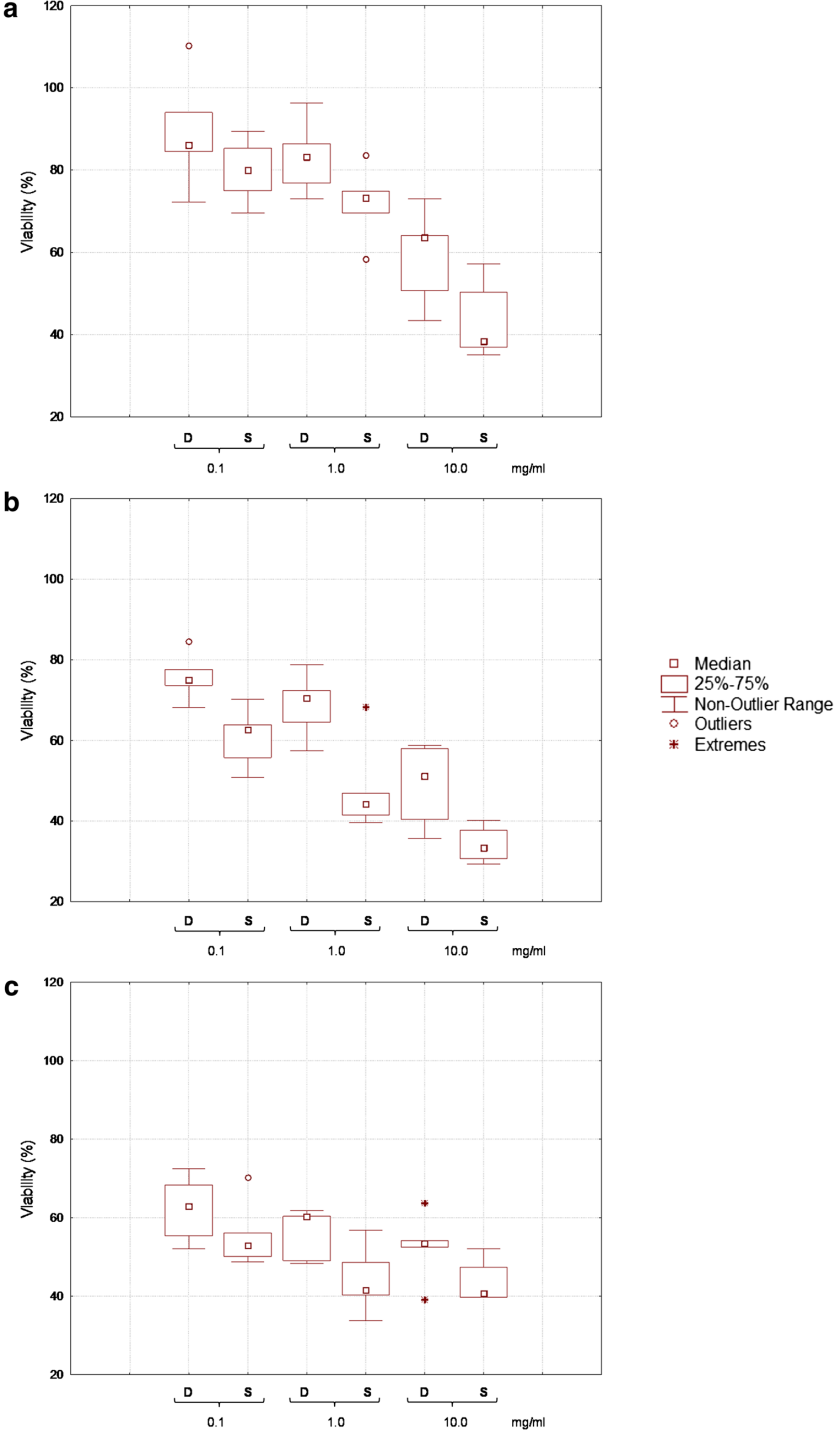
Effect of lunasin-like polypeptides on the Caco-2 cells proliferation. *Box-plot* showing aggregate data for human colon adenocarcinoma (Caco-2) cells proliferation after treatments of 24 (*panel* **a**), 48 (*panel* **b**), and 72 h (*panel* **c**) with water/salt soluble extracts obtained from control doughs (D), without bacterial inoculum, and sourdoughs (S), started with selected lactic acid bacteria, made with Italian legume flours. Protein concentration of 0.1, 1.0, and 10 mg/ml were assayed. Data were expressed as the mean percentage of viable cells compared to the control culture, grown in basal media without the addition of the WSE. The *centre line* of the *box* represents the median (*open square*), the *top* and *bottom* of the *box* represent the 75th and 25th percentile of the data, respectively. The *top* and *bottom* of the *bars* represent the 5th and 95th percentile of the data, respectively. Outliers (*open circle*) and extremes (*aterisk*) are represented

Overall, all the WSE from D and S allowed a significant (*P* < 0.05) decrease of the cell proliferation compared to control (Fig. 2). Compared to the results obtained after 24 h-treatment, the inhibitory effect increased after prolonging the incubation to 48 h, and in many cases, to 72 h. Exceptions were the treatments with 10 mg/ml of proteins, which did not cause a further significant decrease of proliferation. The cytotoxic effect of WSE from S was in all the cases higher than the corresponding D. Besides the effect of the treatment duration, cytotoxicity increased proportionally to the concentration of proteins tested.

After 24 h of treatment (Fig. 2a), a weak anti-proliferative effect was found for WSE from D and S at 0.1 mg/ml of proteins. The strongest effect corresponded to LN: (72.25 ± 2.12 and 69.54 ± 1.88 % of vitality for D and S, respectively. When WSE from S were used at concentration of 1 mg/ml, the vitality of Caco-2 cells decreased by ca. 10 %. In particular, WSE from CC sourdough caused the highest decrease of vitality (58.47 ± 2.08 %). At the highest concentration assayed, the most remarkable cytotoxicity was found for CC, LN, and PS sourdoughs (30.49 ± 1.13, 36.97 ± 1.01, and 35.13 ± 0.95 %, respectively). The same trend was found after 48 h of treatment (Fig. 2b), although lower values of vitality were observed. The lowest proliferation was found for CC sourdough, when treatments were done with 1 mg/ml (39.59 ± 1.05 %), and for CC, LN, and PS sourdoughs, when 10 mg/ml of proteins (30.67 ± 0.87, 29.32 ± 1.11, and 37.75 ± 1.23 %, respectively) were used.

After 72 h of treatment (Fig. 2c), the vitality of Caco-2 cells was lower than that found after 24 or 48 h for treatments performed with 0.1 and 1 mg/ml of proteins, while the use of 10 mg/ml of proteins was less effective than 48 h-treatments, with the only exception of FL sourdough.

### Transcriptional regulation of filaggrin (FLG) and involucrin (IVL)

**Fig. 3:**
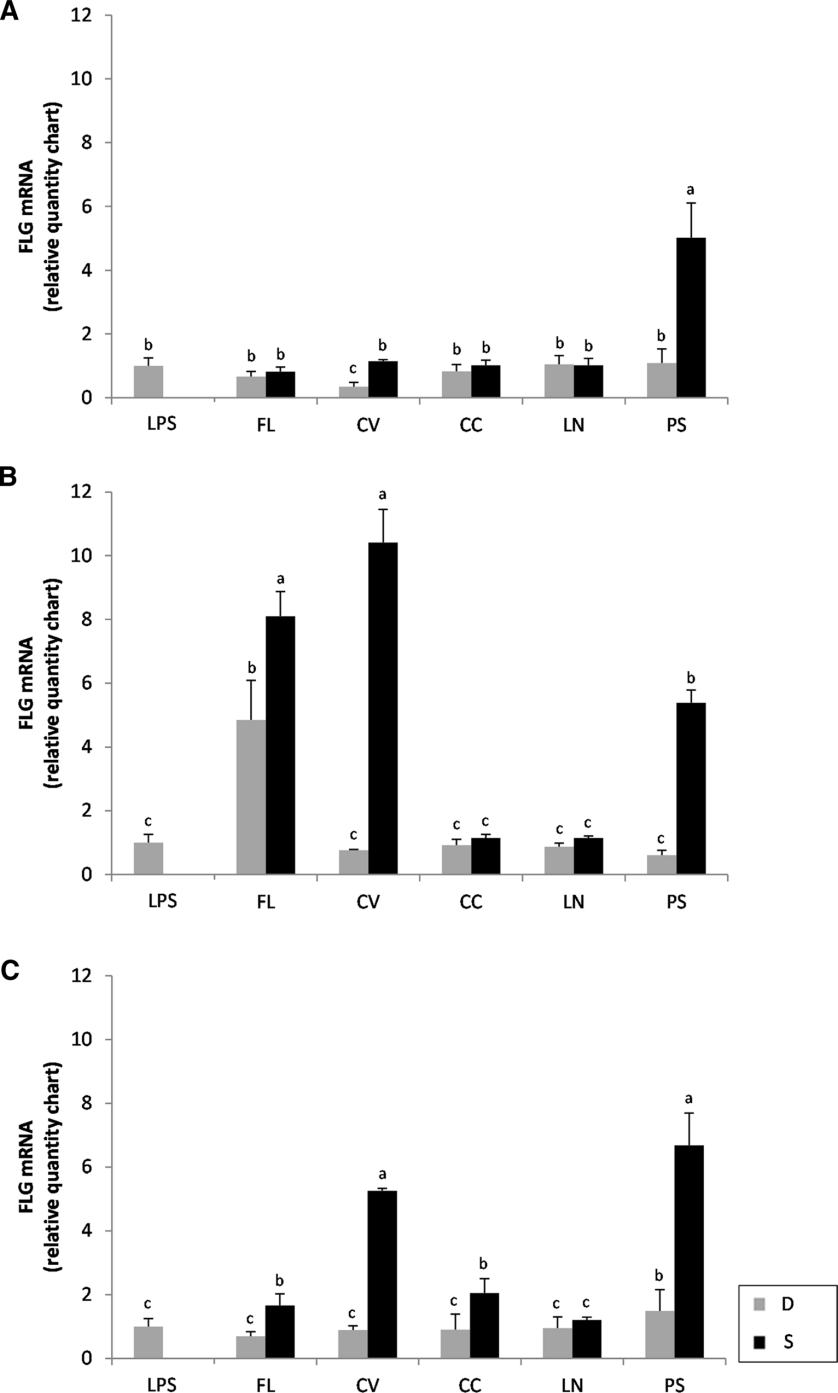
Expression of the filaggrin (*FLG*) gene in Caco-2 cells. The expression of the *FLG* gene in human colon adenocarcinoma cells (Caco-2) was determined using RT-PCR. Caco-2 cells were treated at 37 °C for 4 (**A**), 8 (**B**) and 24 h (**C**) with basal medium containing 1.0 mg/ml of the freeze-dried water/salt soluble extracts obtained from control doughs (D), without bacterial inoculum, and sourdoughs (S), started with selected lactic acid bacteria, made with FL, CV, CC, LN, and PS legume flours. Data are the mean ± SD of three separate experiments, performed in triplicate. *a*–*c* Columns with different *superscript letters* differ significantly (*P* < *0.05*)

Human colon adenocarcinoma cells (Caco-2) were treated with the partially purified and freeze dried WSE from legume D and S aiming at investigating filaggrin (*FLG*) and involucrin (*IVL*) gene expressions through RT-PCR. Treatments were lasting 4, 8, and 24 h, After 4 h of exposure (Fig. 3A), a significant (*P* < 0.05 %) over-expression of FLG was found only for fermented PS. All the other WSE, both from D and S, did not cause significant (*P* < 0.05) variations compared to LPS (positive control). After 8 h of exposure, a significant (*P* < 0.05) up-regulation of the *FLG* gene was also found for FL dough and FL, CV, and PS sourdoughs (Fig. 3B). The same trend was found for treatment lasting 24 h (Fig. 3C) with FL, CV, CC, and PS sourdoughs.

**Fig. 4:**
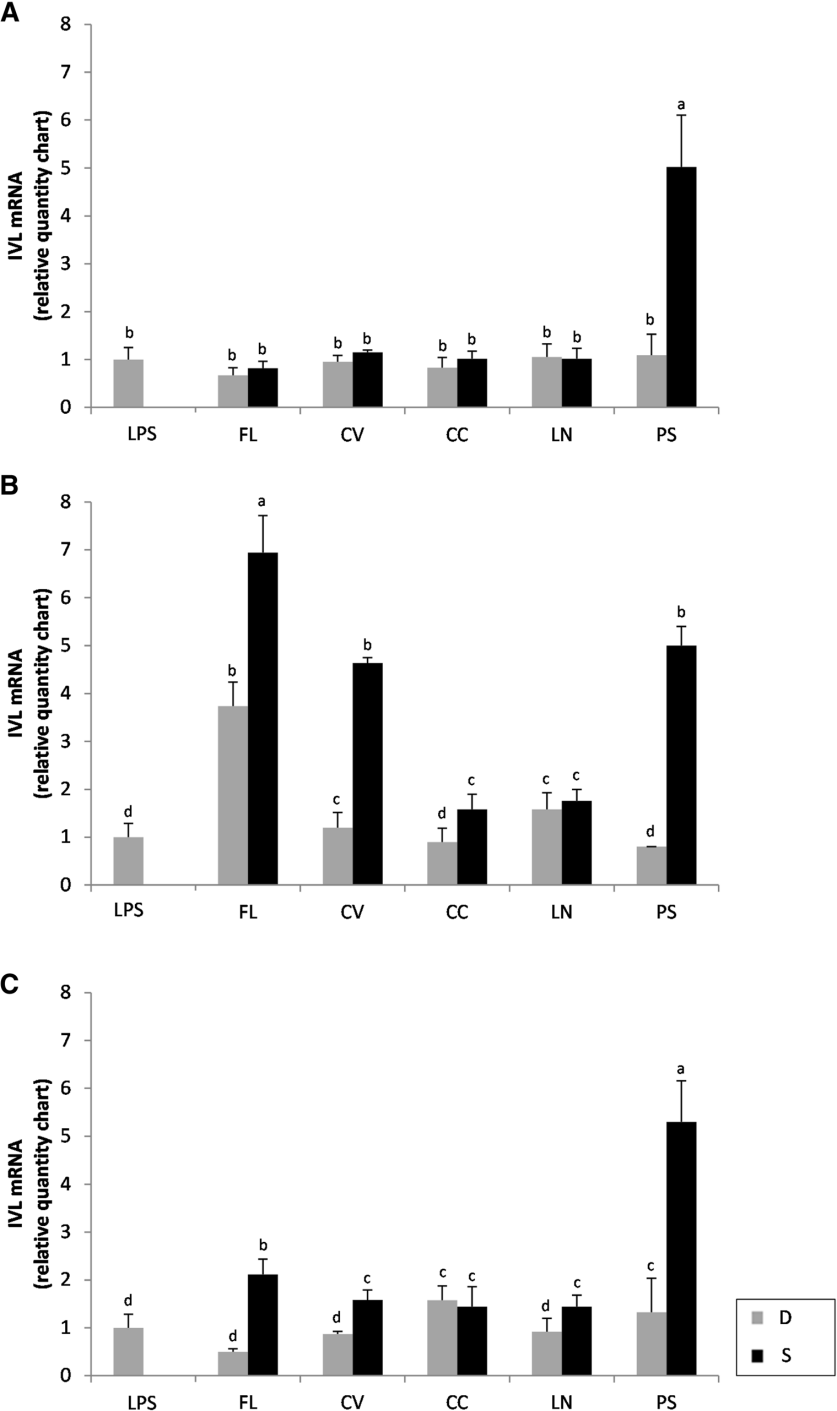
Expression of the involucrin (*IVL*) gene in Caco-2 cells. The expression of the *IVL* gene in human colon adenocarcinoma cells (Caco-2) was determined using RT-PCR. Caco-2 cells were treated at 37 °C for 4 (**A**), 8 (**B**) and 24 h (**C**) with basal medium containing 1.0 mg/ml of the freeze-dried water/salt soluble extracts obtained from control doughs (D), without bacterial inoculum, and sourdoughs (S), started with selected lactic acid bacteria, made with FL, CV, CC, LN, and PS legume flours. Data are the mean ± SD of three separate experiments, performed in triplicate. *a*–*c* Columns with different *superscript letters* differ significantly (*P* < *0.05*)

Regarding *IVL* gene, none of the extracts, except for PS sourdough, induced an expression after 4 h of treatment higher than that of LPS after 4 h of treatment (Fig. 4A). After 8 h of treatment, WSE extracted from S caused significant (*P* < 0.05) increases of the expression compared to the corresponding D, especially FL, CV, and PS (Fig. 4B). A longer treatment with the WSE (24 h) did not favour a further increase of the gene expression. Only PS sourdough caused the same expression of IVL gene under all the conditions assayed (Fig. 4C).

### Identification of lunasin-like polypeptides

Based on the results from the MTT assay on Caco-2 cell, the immunoreactive protein bands of FL, CV, and PS sourdoughs were recovered from Tris-Tricine gels, and subjected to tryptic digestion, and HPLC coupled to nanoESI-MS/MS analysis.

Ten different proteins were identified through the Mascot research on the NCBI database (Table 5). It can be hypothesized that lunasin-like polypeptides were released from native legume proteins via proteolysis during sourdough fermentation. This should explain the lack of correspondence between the molecular masses of the proteins identified and the polypeptide bands revealed by western blot analyses. The immunodetected polypeptide bands from FL sourdough corresponded to the following matches: Subtilisin inhibitor 1 (P16064), Legumin A2 (P15838) and Phaseolin (P02853). The protein bands from CV sourdough matched with: leucoagglutinating phytohemagglutinin (P05087), phatogenesis related protein (CAA56142) and seed linoleate 9S-lipoxygenase-3 (P09918). The proteins from PS sourdough were identified as: Provicilin (P02855), seed linoleate 9S-lipoxygenase-2 (P14856), seed biotin-containing protein SBP65 (Q41060) and Albumin-1 C (P62928). All the database matches corresponded to proteins previously identified from legumes. For five proteins, the exact correspondence between the species analyzed and the database matches was not found, especially for CV. Probably, this was due to the limited number of legume protein sequences previously identified and included in the NCBI database.

**Table 5:** Lunasin-like polypeptides sequences

WSE	Protein	Sequence^a^	NCBI accession number	Theoretical mass (kDa)	Sequence coverage	Mascot score
FL-S	Subtilisin inhibitor 1 (*Vigna angularis*)	QEQGTNPSQEQNVPLPRNYKQALETNTPTKTSWPELVGVTAEQAETKIKEEMVDVQIQVSPHDSFVTADYNPKRVRLYVDESNKVTRTPSIG	P16064	10.38	82	114
	Legumin A2 (*Pisum sativum*)	MATKLLALSLSFCFLLLGGCFALREQPEQNECQLERLNALEPDNRIESEGGLIETWNPNNKQFRCAGVALSRATLQHNALRRPYYSNAPQEIFIQQGNGYFGMVFPGCPETFEEPQESEQGEGRRYRDRHQKVNRFREGDIIAVPTGIVFWMYNDQDTPVIAVSLTDIRSSNNQLDQMPRRFYLAGNHEQEFLRYQHQQGGKQEQENEGNNIFSGFKRDFLEDAFNVNRHIVDRLQGRNEDEEKGAIVKVKGGLSIISPPEKQARHQRGSRQEEDEDEDEERQPRHQRGSRQEEEEDEDEERQPRHQRRRGEEEEEDKKERRGSQKGKSRRQGDNGLEETVCTAKLRLNIGPSSSPDIYNPEAGRIKTVTSLDLPVLRWLKLSAEHGSLHKNAMFVPHYNLNANSIIYALKGRARLQVVNCNGNTVFDGELEAGRALTVPQNYAVAAKSLSDRFSYVAFKTNDRAGIARLAGTSSVINNLPLDVVAATFNLQRNEARQLKSNNPFKFLVPARQSENRASA	P15838	59.63	62	410
	Phaseolin (*Phaseolus vulgaris*)	MMRARVPLLLLGILFLASLSASFATSLREEEESQDNPFYFNSDNSWNTLFKNQYGHIRVLQRFDQQSKRLQNLEDYRLVEFRSKPETLLLPQQADAELLLVVRSGSAILVLVKPDDRREYFFLTSDNPIFSDHQKIPAGIFYLVNPDPKEDLRIIQLAMPVNNPQIHEFFLSSTEAQQSYLQEFSKHILEASFNSKFEEINRVLFEEEGQQEGVIVNIDSEQIKELSKHAKSSSRKSLSKQDNTIGNEFGNLTERTDNSLNVLISSIEMEEGALFVPHYYSKAIVILVVNEGEAHVELVGPKGNKETLEYESYRAELSKDDVFVIPAAYPVAIKATSNVNFTGFGINANNNNRNLLAGKTDNVISSIGRALDGKDVLGLTFSGSGDEVMKLINKQSGSYFVDAHHHQQEQQKGRKGAFVY	P02853	47.540	75	515
CV-S	Leucoagglutinating phytohemagglutinin (*Phaseolus vulgaris*)	MASSKFFTVLFLVLLTHANSSNDIYFNFQRFNETNLILQRDASVSSSGQLRLTNLNGNGEPRVGSLGRAFYSAPIQIWDNTTGTVASFATSFTFNIQVPNNAGPADGLAFALVPVGSQPKDKGGFLGLFDGSNSNFHTVAVEFDTLYNKDWDPTERHIGIDVNSIRSIKTTRWDFVNGENAEVLITYDSSTNLLVASLVYPSQKTSFIVSDTVDLKSVLPEWVSVGFSATTGINKGNVETNDVLSWSFASKLSDGTTSEGLNLANLVLNKIL	P05087	29.54	61	506
	Pathogenesis related protein (*Cicer arietinum*)	MGVFTFEQETASTVPPAKLYKAMVKDADVIIPKAVDAIKTVETVEGNGGPGTIKKLTFVEGGQTLYVLHKIEAIDEANLGYNYSIVGGAGLSETVERYHFEAKLCEGPNGGSIGKVSVKYQTKGDAKPNEKEVQEGKAKGDALFKAIEGYVLANPNYN	CAA56142	16.93	83	408
	Seed linoleate 9S-lipoxygenase-3 (*Pisum sativum*)	MFSGVTGILNRGHKIKGTVVLMRKNVLDINSLTTVGGVIGQGFDILGSTVDNLTAFLGRSVSLQLISATKPDATGKGKLGKATFLEGIISSLPTLGAGQSAFKIHFEWDDDMGIPGAFYIKNFMQTEFFLVSLTLDDIPNHGSIYFVCNSWIYNAKHHKIDRIFFANQTYLPSETPAPLVHYREEELNNLRGDGTGERKEWERIYDYDVYNDLGNPDSGENHARPVLGGSETYPYPRRGRTGRKPTRKDPNSESRSDYVYLPRDEAFGHLKSSDFLTYGLKAVSQNVVPALESVFFDLNFTPNEFDSFDEVHGLYEGGIKLPTNILSQISPLPVLKEIFRTDGENTLKYPPPKVIQVSRSGWMTDEEFAREMLAGVNPNVICCLQEFPPRSKLDSQIYGDHTSKISKEHLEPNLEGLTVEEAIQNKKLFLLDHHDSIMPYLRRINSTSTKAYATRTILFLNNNQNLKPLAIELSLPHPQGDEHGAVSYVYQPALEGVESSIWLLAKAYVIVNDSCYHQLVSHWLNTHAVVEPFVIATNRHLSCLHPIYKLLYPHYRDTMNINSLARLSLVNDGGIIEKTFLWGRYSMEMSSKVYKNWVFTEQALPADLIKRGMAIEDPSSPCGVKLVVEDYPYAVDGLEIWAIIKTWVQDYVSLYYTSDEKLRQDSELQAWWKELVEVGHGDKKNEPWWPKMQTREDLIEVCSIVIWTASALHAAVNFGQYSYGGLILNRPTLSRRFMPEKGSAEFEELVKSPQKAYLKTITPKFQTLIDLSVIEILSRHASDELYLGERDNPNWTSDKRALEAFKKFGNKLAEIEKKLTQRNNDEKLRNRHGPVEMPYTLLYPSSKEGLTFRGIPNSISI	P09918	97.57	43	207
PS-S	Provicilin (*Pisum sativum*)	DNAEIEKILLEEHEKETHHRRGLRDKRQQSQEKNVIVKVSKKQIEELSKNAKSSSKKSVSSRSEPFNLKSSDPIYSNQYGKFFEITPKKNPQLQDLDIFVNYVEIKEGSLWLPHYNSRAIVIVTVNEGKGDFELVGQRNENQQGLREEDDEEEEQREEETKNQVQSYKAKLTPGDVFVIPAGHPVAVRASSNLNLLGFGINAENNQRNFLAGEEDNVISQIQKQVKDLTFPGSAQEVDRLLENQKQSYFANAQPQQRETRSQEIKEHLYSILGAF	P02855	31.52	53	589
	Seed linoleate 9S-lipoxygenase-2 (*Pisum sativum*)	MFPNVTGLLNKGHKIRGTVVLMRKNVLDFNTIVSIGGGNVHGVIDSGINIIGSTLDGLTAFLGRSVSLQLISATKSDANGKGKVGKDTFLEGVLASLPTLGAGESAFNIHFEWDHEMGIP121GAFYIKNYMQVEFFLKSLTLEDVPNHGTIRFVCNSWVYNSKLYKSPRIFFANKSYLPSETPSPLVKYREEELQTLRGDGTGERKLHERIYDYDVYNDLGNPDHGEHLARPILGGSSTHPYPRRGRTGRYPTRKDPNSEKPATETYVPRDENFGHLKSSDFLAYGIKSVSQCVVPAFESAFDLNFTPNEFDSFQDVRNLFEGGIKLPLDVISTLSPLPVVKEIFRTDGEQVLKFTPPHVIRVSKSAWMTDEEFAREMLAGVNPCMIRGLQEFPPKSNLDPAEYGDHTSKISVDVLNLDGCTIDEALASGRLFILDYHDTFIPFLRRINETSAKAYATRTILFLKENGTLKPVAIELSLPHPDGDKSGFVSKVILPADEGVESTIWLLAKAYVVVNDSCYHQLMSHWLNTHAVIEPFVIATNRQLSVVHPINKLLAPHYRDTMMNINALARDSLINANGLIERSFLPSKYAVEMSSAVYKYWVFTDQALPNDLIKRNMAVKDSSSPYGLRLLIEDYPYAVDGLEIWTAIKTWVQDYVSLYYATDNDIKNDSELQHWWKEVVEKGHGDLKDKPWWPKLQTFDELVEVCTIIIWTASALHAAVNFGQYPYGGLILNRPTLSRRLLPEEGTAEYDEMVKSSQKAYLRTITPKFQTLIDLSVIEILSRHASDEVYLGQRENPHWTSDSKALQAFQKFGNKLAEIEAKLTNKNNDPSLYHRVGPVQLPYTLLHPSSKEGLTFRGIPNSISI	P14856	97.07	36	178
	Seed biotin-containing protein SBP65 (*Pisum sativum*)	MASEQLSRRENITTERKIQNAEDSVPQRTTHFELRETHELGPNFQSLPRNENQAYLDRGARAPLSANVSESYLDRARVPLNANIPEHRVREKEDFGGVRDMGKFQMESKGGNKSLAEDRETLDTRSRMVTGTPHIKEASGKGQVVEERERARERAMEEEEKRLTMEEISKYRNQAQQSALEALSAAQEKYERAKQATNETLRNTTQAAQEKGEAAQAKDATFEKTQQGYEMTGDTVSNSARTASEKAAQAKNTTLGKTQQGYEATRDTVSNAARTAAEYATPAAEKARCVAVQAKDVTLETGKTAAEKAKCAAEIAAKVAVDLKEKATVAGWTASHYATQLTVDGTRAAANAVEGAVGYVAPKASELAAKSVETVKGLAASAGETAKEFTARKKEESWREYEAKRASQLQEGEEILPSTGGIGKVLPSGERTQAQGTNLQEKVQGKGSDILGAVTETVSDIGSSMIKPIDNANTKVKEHGGTTITPKGQDAGGVLDAIGETIAEIAHTTKVIVVGEDDEVEKSMQKNIGSDSHSLDRAKHEGYRAPKNNVS	Q41060	59.52	46	783
	Albumin-1 C (*Pisum sativum*)	MASVKLASLIVLFATLGMFLTKNVGAISCNGVCSPFDIPPCGSPLCRCIPAGLVIGNCRNPYGVFLRTNDEHPNLCESDADCRKKGSGTFCGHYPNPDIEYGWCFASKSEAEDVFSKITPKDLLKSVSTA	P62928	13.90	55	333

Identification was carried out by nano-LC–ESI–MS–MS. Polypeptides were purified from water/salt-soluble extracts obtained from sourdoughs (-S) made with Fagiolo di Lamon (FL), Cece dell’Alta Valle del Misa (CV), and Pisello riccio di Sannicola (PS)

^a^Single-letter amino acid code is used

**Fig. 5:**
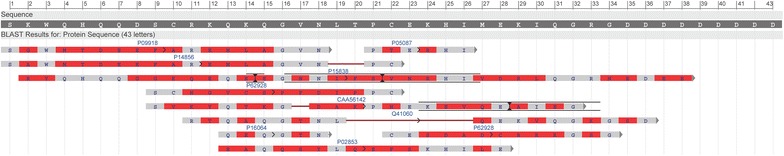
Alignments with lunasin. Alignments of the lunasin-like polypeptides identified from FL, CV, and PS sourdoughs with the soy lunasin sequence, as obtained through the BLAST alignment on-line tool (http://blast.ncbi.nlm.nih.gov/)

The protein sequences were compared to soy lunasin sequence (deposited at the National Center for Biotechnology Information, NCBI database with the accession number AAP62458) using BLAST. Except for Provicilin identified that was identified in PS sourdough, all the sequences might be aligned with different lunasin epitopes (Fig. 5). The longest alignments were found for Legumin A2, corresponding to fragments (f) 24–38 and 33–41 of the soy lunasin sequence, and Phaseolin (f13–28) from FL sourdough; pathogenesis related protein and seed-linoleate 9S lipoxygenate-3 (f9–32 and f1–18, respectively) from CV, and seed-linoleate 9S lipoxygenate-2 and seed biotin-containing protein SBP65 (f1–22 and f11–36, respectively) from PS. The alignments showed identities from 26 to 67 % (pathogenesis related protein and subtilisin inhibitor1/leucoagglutinating phytoemagglutinin, respectively) and positives from 35 to 93 % (Albumin-1C and Legumin A2, respectively).

### Peptidase activities

The peptidase activities of the two lactic acid bacteria strains used as starters were assayed using relatively specific synthetic substrates. Significant (*P* < 0.05) differences were found between strains. Compared to that of *L. plantarum* C48 (5.87 ± 0.02 and 3.52 ± 0.02 U), PepN activity on Leu-*p*-NA and endopeptidase activity (PepO) of *L. brevis* AM7 were higher (6.83 ± 0.08 U and 4.31 ± 0.03). Also PepA activity was higher in *L. brevis* AM7 (14.02 ± 0.04 U). That of *L. plantarum* C48 resulted 35 % lower. This latter strain showed higher tripeptidase (PepT) activity (9.12 ± 0.02 vs. 5.61 ± 0.05 U). No significant differences (*P* > *0.05*) were found between the two strains for PepT (7.20 ± 0.04 and 7.12 ± 0.05 U) and PepX (0.95 ± 0.03 and 1.01 ± 0.02 U) activities.

## Discussion

Despite the beneficial effects on the human diet, the worldwide consumption of legumes is declining [15] and below the recommended dose [16]. One potential way to increase the consumption of legumes could be the rediscovery of traditional and local varieties, and, especially, the use of legumes into novel and healthy foods, also exploiting the potential of non-conventional processing [17]. The complementation between cereal and legume flours into new formulas may deserve an interest either to increase the levels of biogenic compounds or to fulfill nutritional deficiencies of cereal-based diets [18].

Traditional Italian legumes, all with product certifications and belonging to *Phaseulus vulgaris*, *Cicer arietinum*, *Lathyrus sativus*, *Lens culinaris* and *Pisum sativum* species, were used in this study. Seeds were milled, and flours were subjected to sourdough fermentation, using the selected *Lactobacillus plantarum* C48 and *Lactobacillus brevis* AM7.

Compared to cereals, all legume flours showed elevated amount of protein (mainly grass pea and several lentil flours), FAA (>2 g/kg), dietary fibers, and ash. In particular, grass pea varieties had the highest concentration of proteins, while pea, chickpea, and grass pea flours contained the highest levels of dietary fibre. The microbiota of legume flours was poorly represented by lactic acid bacteria and yeasts. All the data agreed with previous findings [19].

Recently, the potential of sourdough fermentation was exploited to enhance the nutritional and functional features of legume flours [19]. Apart from the legume species and variety, sourdough fermentation with selected starters is a suitable biotechnology option either to increase the nutritional and functional value or to decrease the levels of anti-nutritional factors [19]. Selected sourdough lactic acid bacteria were able to decrease the concentration of raffinose up to ca. 64 % and a similar trend was found for the concentration of condensed tannins [19]. The sourdough fermentation also increased the concentration of GABA, and promoted antioxidant and phytase activities compared to the raw flours [19].

According to protocols used for cereal sourdough fermentation [20], legume flours were started with *Lactobacillus brevis* AM7 and *Lactobacillus plantarum* C48 [21]. As previously shown [19], the conditions of incubation (24 h at 30 °C) allowed the optimal growth and metabolism of selected lactic acid bacteria [19, 22]. As expected, fermentation caused a marked decrease of the values of pH as well as an increase of TTA and of the concentration of peptides and FAA, especially when kidney bean flours were used.

Based on their proteolytic activity towards vegetable proteins [20], the use of sourdough lactic acid bacteria for synthesizing bioactive peptides deserves a marked interest [23]. Bioactive peptides derived from food proteins may possess physiological properties beyond the role in nutrition. These properties are influenced by the protein source, enzyme and processing conditions used [24]. Most of the research related to bioactive peptides and cancer was focused on lunasin [25] and, recently, on pulse hydrolysates [2].

To the best of our knowledge, no literature data dealt with the presence of lunasin or lunasin-like polypeptides from native protein sequences of Italian pulses, and with the effect of sourdough fermentation on bioactivity and bioavailability.

Proteinase activity and a large portfolio of peptidases are the pre-requisites to liberate bioactive peptides from native oligopeptides [7, 26]. Although with some differences, the two starters used showed different peptidase activities.

Western blot analyses showed that the sequence of lunasin sequence was absent in all the legume flours, and, therefore, in the corresponding sourdoughs. Nevertheless, immunoreactive polypeptides with molecular masses lower than 30 kDa were detectable in all the samples. In some cases, immunoreactive polypeptides appeared as multiple bands in western blot, probably due to the presence of multiple fragments differing for few aminoacid residues [12]. Regarding unfermented doughs, proteins with high intensity signals were found for all the bean, followed by lentil varieties. After lactic acid bacteria fermentation, the number and the intensity of the proteins reacting with the anti-lunasin antibody increased for all the legume flours. This was probably due to an acid activation of endogenous proteinases, responsible for primary proteolysis, and peptidase activities by lactic acid bacteria, which completed the hydrolysis (secondary proteolysis). All the reactive protein bands had molecular masses higher than that of lunasin. Although some differences were found among varieties, one representative was chosen for each legume species to assay the effect on Caco-2 cells proliferation. Caco-2 cells derived from a colonic tumor and have a cancerous phenotype, and can be cultivated to become confluent. In this case, they differentiate into enterocyte-like cells [27].

Polypeptides able to regulate cell proliferation and survival were already liberated by enzyme hydrolysis of plant and animal proteins [28]. Such peptides, inhibiting cell growth or promoting apoptosis, could have protective effects on tumor growth at the digestive tract level [29, 30]. In particular, it was found that the antitumoral mechanism of soybean lunasin is related to the capacity of the peptide to bind the deacetylated histones, causing the acetylation inhibition [31, 32]. Girón-Calle et al. [28] assessed the cancer cell proliferation after treatment with chickpea hydrolysates made with pepsin/pancreatin. These hydrolysates inhibited the proliferation of human epithelial colorectal adenocarcinoma cells (Caco-2) and monocytics leukemia cells (THP-1) up to 48 and 78 %, respectively. Hydrolysates from the common bean (*P. vulgaris*) varieties Negro 8025 and Pinto Durango inhibited the inflammation in lipopolysaccharide-induced macrophages through suppression of NF-κB pathways [2]. After hydrolysis with Alcalase, proteins extracted from both the above varieties inhibited various markers of inflammation (cyclooxygenase-2 expression, prostaglandin E2 production, inducible nitric oxide synthase expression, and nitric oxide production) [2]. Inflammation and cancer are linked, chronic inflammation predisposes individuals to various types of cancer and inflammatory mediators and cells are involved in the migration, invasion, and metastasis of malignant cells [33]. The suppression of pro-inflammatory pathways may provide opportunities for both prevention and treatment of cancer [34].

In this study, extracts from legume doughs regulated the proliferation of Caco-2 cells, including those obtained from control doughs, in which very weak signals of immunoreactive bands were found with the western blot analysis. It can be hypothesized that the effect could be related to the contribution of some legume proteins or peptides able to act as anticancer compounds in their native form, causing cytotoxicity and apoptosis of tumoral cells [2, 5]. Nevertheless, a strong inhibition of the Caco-2 cell proliferation was found only after lactic acid fermentation. A marked inhibition was found when cells were treated for 24 h with the fermented legume extracts at concentrations from 1 to 10 mg/ml. Sourdoughs from FL, CV, and PS flours were the most active, showing a decrease of Caco-2 cells vitality up to 70 %. A correlation between the anti-proliferative effect of protein hydrolysates towards Caco-2 cells and the in vivo growth inhibition of tumors in the digestive tract was demonstrated [29, 30].

The nanoESI-MS/MS spectrometer analysis of the immunoreactive protein bands from water/salt-soluble extracts of FL, CV and PS sourdoughs allowed the identification of ten legume proteins. According to the molecular masses of the identified proteins, it can be hypothesized that immunoreactive polypeptides are encrypted into the native sequences and released as fragments during lactic acid bacteria fermentation. Nine of them showed similarities to soy lunasin sequence, probably related to their recognition by the anti-lunasin antibody.

Using Caco-2 cells, the capacity to induce the expression of human *FLG* and *IVL* genes by sourdough fermented legumes was investigated. FLG and IVL are important proteins for the formation of the epidermal skin barrier [35, 36]. FLG aggregates keratin filaments and provides a cytoskeleton for the cornified envelope [35]. The expression of FLG was markedly induced by PS sourdough, and under some of the assayed conditions (e.g., 8 h of treatment), also by CV and FL sourdoughs. Compared to the unfermented dough, the level of IVL gene expression markedly increased when Caco-2 cells were subjected to 24-h treatment with FL, CV, and PS sourdoughs. In particular, the sourdough made with PS was effective under all the conditions likely it was observed for FLG gene expression. IVL serves as a substrate for the covalent attachment of ceramides to the cornified envelope [35, 36]. An improvement of the IVL activity may result in an increase of the ceramide binding activity, which, in turn, positively affects the barrier function [37, 38].

## Conclusions

Nowadays, an unexpected and considerable number of small proteins and peptides are available as drugs [39]. They show high bioactivity, target specificity and wide spectrum of therapeutic actions, with low levels of toxicity, structural diversity, and absence or low levels of accumulation in body tissues. However, the manufacturing protocols (e.g. chemical synthesis and recombinant transgenic approach) are very expensive, representing a hindrance for the use as therapeutic peptides [40]. The alternative option is the exploitation of the potential of bioactive peptides derived from food proteins. Legume flours were already proposed as adjuvant ingredients into a range of baked products and snacks for increasing the nutritional value [41–44], as well as sourdough fermentation was shown to have a functional potential [19, 45, 46]. Despite the need to have an in vivo confirmation, the presence of lunasin-like polypeptides allows to hypothesize the use of sourdough fermented legumes in pharmaceuticals preparations (e.g., capsules and powders), protein hydrolysates or as purified peptide mixtures to be incorporated as health-enhancing ingredients in novel functional foods (e.g., leavened baked goods).

## Methods

### Legumes

Nineteen Italian legume varieties, belonging to the species *Phaseulus vulgaris* (Fagiolo di Lamon, Fagiolo di Controne, Fagiolo di Cuneo, Fagiolo Stregoni, Fagiolo Vellutina, Fagiolo di Saluggia, Fagiolo Badda di Polizzi—white, and Fagiolo Badda di Polizzi—black), *Cicer arietinum* (Cece di Merella and Cece dell’Alta Valle del Misa), *Lathyrus sativus* (Cicerchia di Serra de Conti and Cicerchia di Campodimele), *Lens culinaris* (Lenticchia di Castelluccio di Norcia, Lenticchia di Ustica, Lenticchia di Santo Stefano di Sessanio, Lenticchia rossa di Pantelleria, Lenticchia di Altamura and Lenticchia di Villalba), and *Pisum sativum* (Pisello riccio di Sannicola), were used in this study. All legumes were chosen among the Italian pulses that have specific product certification (Table 1). Flours were obtained from whole legume seeds through the laboratory mill Ika-Werke M20 (GMBH, and Co. KG, Staufen, Germany). Protein (total nitrogen ×5.7), ash and moisture contents were determined according to AACC approved methods 46-11A, 08-01 and 44-15A, respectively [47]. Lipids were determined by Soxhlet method. Total carbohydrates were calculated as the difference, using the following formula: [100 − (proteins + lipids + ash + moisture)]. Proteins, lipids, carbohydrates and ash were expressed as % of dry matter (d.m.). The determination of total dietary fiber was carried out by the enzymatic–gravimetric procedure approved by the Association of Official Analytical Chemists [48], as described by Lee et al. [49].

### Microbiological analyses of the flours

Ten grams of flour were homogenized with 90 ml of sterile peptone water [1 % (wt/vol) of peptone and 0.9 % (wt/vol) of NaCl] solution. Lactic acid bacteria were enumerated using MRS (Oxoid, Basingstoke, Hampshire, UK) agar medium, supplemented with cycloheximide (0.1 g/l). Plates were incubated, under anaerobiosis (AnaeroGen and AnaeroJar, Oxoid), at 30 °C for 48 h. Cell density of yeasts and molds were estimated on Yeast Extract Peptone Dextrose Agar (YPD) (Oxoid) medium (pour and spread plate enumeration, respectively), supplemented with chloramphenicol (0.15 g/l), at 30 °C for 72 h. The attribution (yeasts/molds) was confirmed by microscope observation. Total mesophilic aerobic bacteria were determined on Plate Count Agar (PCA, Oxoid) at 30 °C for 48 h, and total enterobacteria were determined on Violet Red Bile Glucose Agar (VRBGA, Oxoid) at 37 °C for 24 h.

### Fermentation

Sourdough lactic acid bacteria, belonging to the Culture Collection of the Department of Soil, Plant, and Food Sciences (University of Bari, IT) and previously selected for some biotechnological features, were used. *Lactobacillus plantarum* C48 showed the capacity of synthesizing relevant amount of γ-aminobutyric acid (GABA) [21], and *Lactobacillus brevis* AM7 was characterized by high proteolytic activities towards cereal and legume flours [12]. Starters were cultivated separately on MRS broth at 30 °C for 24 h. Cells were harvested by centrifugation (10,000×*g*, 10 min, 4 °C) until the late exponential phase of growth was reached (ca. 10 h), washed twice in 50 mM sterile potassium phosphate buffer (pH 7.0) and re-suspended in tap water at the cell density of ca. 8.0 log cfu/ml. Each legume flour was mixed with tap water, containing the bacterial suspension (initial cell density of 7.0 log cfu/g of sourdough for each strain). Doughs, having dough yield (DY, dough weight × 100/flour weight) of 160 (corresponding to 62.5 and 37.5 % of flour and water, respectively), were mixed at 60 *x g* for 5 min with a IM 58 high-speed mixer (Mecnosud, Flumeri, Italy) and incubated at 30 °C for 24 h. The most common DY value for cereal-based sourdoughs (160) was applied [20, 22, 46, 50]. After fermentation, legume sourdoughs (S) were compared to control doughs (D), without bacterial inoculum, prepared as described above (DY 160) and incubated under the same conditions. S and D were stored at −20 °C before the chemical analyses, while microbiological analysis was carried out before freezing. All the doughs were obtained in triplicate and each of them was analyzed twice.

### Characterization of fermented flours

The values of pH were determined on-line by a pHmeter (Model 507, Crison, Milan, Italy) with a food penetration probe. Total titratable acidity (TTA) was determined after homogenization of 10 g of dough with 90 ml of distilled water, and expressed as the amount (ml) of 0.1 M NaOH needed to reach the value of pH of 8.3.

The water/salt-soluble extract (WSE) of D and S was prepared according to Weiss et al. [51], and used to analyze free amino acids (FAA) by a Biochrom 30 series Amino Acid Analyzer (Biochrom Ltd., Cambridge Science Park, UK) with a Na-cation-exchange column (20 by 0.46 cm internal diameter), as described by Rizzello et al. [50].

### Peptidase activities of the starters

General aminopeptidase type N (EC 3.4.11.11; PepN), specific aminopeptidase type A (EC 3.4.11.7; PepA) and endopeptidase (EC 3.4.23; PepO) activities were determined as described by Gobbetti et al. [13], using, respectively, Leu-*p*-nitroanilides (*p*-NA), Glu-*p*-NA and NCBZ-Gly-Gly-Leu-*p*-NA (Sigma Aldrich Co.) as relatively specific substrates. The assay mixture contained 900 μl of 2.0 mM substrate in 0.05 M potassium phosphate buffer, pH 7.0, and 100 μl of cell suspension (9 log cfu/ml). The mixture was incubated at 30 °C for 1 h and the absorbance was measured at 410 nm. The data obtained were compared to standard curves set up by using *p*-nitroanilide [52]. One unit (U) of activity was defined as the amount of enzyme required to liberate 1 μmol/min of *p*-nitroanilide under the assay conditions. Tripeptidase (EC3.4.11.4; PepT), and X-prolyl dipeptidyl aminopeptidase (EC 3.4.14.5; PepX) activities were determined using Leu-Leu-Leu and Gly-Pro-Ala substrates (Sigma Aldrich Co.), respectively. Activities on tripeptides were determined by the Cd-ninhydrin method [52], [53]. The assay conditions were the same as those described for *p*-nitroanilide substrates. One unit (U) of activity was defined as the amount of enzyme required to liberate 1 μmol amino acid released per min under the assay conditions. The data obtained were compared to the standard curve set up by using leucine [52].

### Western blot analysis

Protein concentration of samples was determined by the bicinchoninic acid method (Thermo Scientific, Rockford, IL, USA), using bovine serum albumin (BSA) as standard protein. SDS-PAGE was performed with samples and synthetic lunasin diluted in tricine sample buffer (Bio-Rad, Richmond, CA, USA), containing 2 % (v/v) β-mercaptoethanol, and heated at 100 °C for 5 min. Equal amounts of proteins (20–40 µg) were analyzed on Precast Criterion 16.5 % Tris-Tricine gels (Bio-Rad), and electrophoretic separations were carried out at 100 V, using Tris-Tricine-SDS as running buffer in the Criterion cell (Bio-Rad). For the attribution of the molecular masses, a Precision Plus Protein Standards mix (BioRad) was used. After SDS-PAGE separation, gels were soaked in transfer buffer (48 mM Tris, 39 mM glycine, 20 % methanol, pH 9.2) for 30 min. Proteins were electroblotted into nitrocellulose membranes by semidry transfer in a Trans-Blot SD (Bio-Rad) for 30 min at 18 V. Then, the membranes were blocked for 3 h in Tris buffered saline with 0.05 % (v/v) Tween 20 (TBST), containing 1 % (w/v) bovine serum albumin, (TBST-1 % BSA). Afterwards, the membrane was washed three times with TBST, and incubated overnight at 4 °C with lunasin polyclonal primary antibody (diluted 1:2000 in TBST-0.1 % BSA). After washing with TBST, the membrane was incubated overnight at 4 °C with horseradish peroxidase-conjugated mouse anti-rabbit secondary antibody (Santa Cruz Biotechnology, Dallas, TX, USA; 1:3000 in TBST-0.1 % BSA). Finally, the membranes were washed six times with TBST, and visualized by chemioluminescence using the detection agent Amersham TM ECL Prime (GE Healthcare, Chalfont St Giles, UK), according to the manufacturer´s recommendations. Image acquisition (exposure time 1–4 min) was performed using the VersaDoc Imaging System (Bio-Rad).

### MTT assay in Caco-2 cells

In order to assess the effect of legume D and S on cell proliferation, the viability of colon adenocarcinoma Caco-2 cells was measured using the 3-(4,5-dimethyl-2-yl)-2,5-diphenyltetrazolium bromide (MTT) method [54].

Colon adenocarcinoma Caco-2 cells (ICLC HTL97023), provided by the National Institute for Cancer Research of Genoa (Italy), were cultured in RPMI medium, supplemented with 10 % Fetal Bovine Serum (FBS), 1 % 2 mM l-glutamine, 1 % penicillin (10,000 U/ml)/streptomycin (10,000 μg/ml) mixture, 0.1 % gentamicin and 0.1 % β-mercaptoethanol, and maintained in 25 cm^2^ culture flasks at 37 °C, 5 % CO_2_. Every 2 days, confluent cultures were washed with PBS 1× (without Ca^2+^ and Mg^2+^), split 1:3–1:6 using Trypsin/EDTA, and seeded at 2–5 × 10^4^ cell/cm^2^, 37 °C, and 5 % CO_2_. For the assay, Caco-2 cells were harvested at 80 % confluence with trypsin/EDTA, seeded at the density of 5 × 10^4^ cells/ml into 96-well plates, and then incubated at 37 °C, 5 % CO_2_ for 24 h. Before analysis, WSE obtained from legume D and S were partially purified by ultra-filtration, using Vivaspin (GE Healthcare) centrifugal filter units (cut-off 30 kDa), following the manufacturer’s instructions.

Cells were exposed to partially purified and freeze dried WSE at the following concentrations: 0.1, 1.0, and 10.0 mg/ml of proteins. Each WSE was tested in duplicate. A control in basal medium, without addition of WSE, was used. Incubation was carried out for 24, 48 and 72 h. After incubation, the medium was removed and replaced by 100 µl/well of MTT solution. Then, plates were incubated in the dark for 3 h (37 °C, 5 % CO_2_). MTT salt was dissolved in PBS (5 mg/ml) and added (1:10) to RPMI, containing 10 % FBS, 1 % 2 mM l-glutamine, 1 % penicillin (10,000 U/ml)/streptomycin (10,000 μg/ml) mixture, 0.1 % gentamicin and 0.1 % β-mercaptoethanol. Then, the medium was removed and 100 µl/well of dimethyl sulphoxide (DMSO) were added to dissolve the purple formazan product. Plates were shacked for 15 min at room temperature and the absorbance was read at a wavelength of 570 nm, with a Biotek microplate reader (BioTek Instruments Inc., Bad Friedrichshall, Germany), and elaborated with the ELX808 software (BioTek Instruments Inc., Bad Friedrichshall, Germany). Data were expressed as the mean percentage of viable cells compared to control culture, grown in basal media without addition of WSE.

### Identification of the lunasin-like polypeptides

After Western blot analysis, the immunoreactive bands, separated by Tris-Tricine SDS PAGE, were cut and stored in 20 % ethanol before identification. Protein identification was performed by Proteome Factory (Proteome Factory AG, Berlin, Germany). Protein bands were in-gel digested by trypsin (Promega, Mannheim, Germany) and analyzed by Nano-Liquid Cromatography-Electrospray Ionisation-Mass Spectrometry (nanoLC-ESI–MS/MS). The LC–MS system consisted of an Agilent 1100 nanoHPLC system (Agilent, Waldbronn, Germany), PicoTip electrospray emitter (New Objective, Woburn, MA, USA) and an Orbitrap XL or LTQFT Ultra mass spectrometer (ThermoFisher Scientific, Bremen, Germany). Peptides were first trapped and desalted on the enrichment column (Zorbax 300SB-C18, 0.3 × 5 mm, Agilent) for 5 min (solvent: 2.5 % acetonitrile/0.5 % formic acid), then separated on a Zorbax 300SB-C18, 75 μm × 150 mm column (Agilent), using a linear gradient from 10 to 32 % B (solvent A: 5 % acetonitrile in water, solvent B: acetonitrile, both with 0.1 % formic acid). Ions of interest were data-dependently subjected to MS/MS, according to the expected charge state distribution of peptide ions. Proteins were identified by database search against the plant sequences of the National Center for Biotechnology Information, (NCBInr, Bethesda, USA) protein database, using MS/MS ion search of the Mascot search engine (Matrix Science, London, UK). Only peptide matches with a score of 20 or above were accepted. The sequences of the protein identified were aligned using the BLAST on-line tools (http://blast.ncbi.nlm.nih.gov).

### Transcriptional regulation of filaggrin (*FLG*) and involucrin (*IVL*) genes

Colon adenocarcinoma Caco-2 cells (ICLC HTL97023) were cultured in RPMI medium supplemented with 10 % FBS, 1 % 2 mM l-glutamine, 1 % penicillin (10,000 U/ml)/streptomycin (10,000 μg/ml) mixture, 0.1 % gentamicin and 0.1 % β-mercaptoethanol and maintained in 25 cm^2^ culture flasks at 37 °C, 5 % CO_2_. Caco-2 cells were incubated in 25-cm^2^ culture flasks at 37 °C, under 5 % CO_2_ atmosphere [55].

Subconfluent monolayers of Caco-2 cells were subjected to treatment with basal medium, containing 2.5 % FBS. The freeze-dried WSE, partially purified by ultra-filtration using Vivaspin (GE Healthcare) centrifugal filter units (cut-off 30 kDa), were dissolved in RPMI medium, at the concentration of 10 mg/ml and added to the culture media at the final concentration of 1 mg/ml. The control was the basal medium, containing 2.5 % FBS. Plates were incubated at 37 °C for 24 h, under 5 % CO_2_. Samples were taken after 4, 8, and 24 h of treatment. Each experiment was carried out at least twice in triplicate. For quantitative real-time PCR (RT-PCR), total RNA was extracted from Caco-2 cells using the Ribospin Minikit-GeneAll kit. cDNA was synthesized from 2 µg RNA template in a 20-µl reaction volume, using the Prime Script RT reagent kit (perfect Real time) (Takara). Total RNA solution (10 µl) was added to the Master Mix and subjected to reverse transcription in a thermal cycler (Stratagene Mx3000P Real-time PCR System, Agilent Technologies Italia Spa, Milan, Italy). The conditions were 37 °C for 15 min, 85 °C for 5 s, holding the samples at 25 °C.

The cDNA was amplified and detected using the same instrument and the TaqMan assay (Applied Biosystems). The following Taqman gene expression assays were used: FLG Hs00863478_g1 (FLG), IVL Hs00846307_s1 (IVL), and GAPDH Hs99999905_m1 (human glyceraldehyde-3-phosphate dehydrogenase, GAPDH). Human GAPDH was used as the housekeeping gene. PCR amplifications were carried out using 40 ng cDNA in a total volume of 20 µl. The reaction mixture contained 10 μL of Premix Ex Taq, 0.4 μl of RoxTM reference dye II, 1 μL of 20X TaqMan Gene Expression assay and 4 µl of cDNA.

PCR conditions were 95 °C for 30 s (for AmpliTaq activation), followed by 40 amplification cycles (95 °C for 5 s, 60 °C for 20 s). Analyses were carried out in triplicate. Based on preliminary results, the expression of FLG and IVL genes was also assayed by treating Caco-2 cells with lipopolysaccharide (LPS; Sigma Aldrich Co.) at 10 µg/ml in basal medium, containing 2.5 % FBS. Analyses were carried out in triplicate. The average value of target gene was normalized using *GAPDH* gene and the values were elaborated automatically by the MXPro v.4.01 Stratagene software [56].

### Statistical analysis

Data were subjected to one-way ANOVA; pair-comparison of treatment means was achieved by Tukey’s procedure at *P* < 0.05, using the statistical software, Statistica 7.0 for Windows. Student’s *t* test was used for MTT assay (GraphPAD 6.0 for Windows).

## Abbreviations

BSA: bovine serum albumin
D: dough
DY: dough yield
FBS: fetal bovine serum
FLG: filaggrin
GABA: γ-aminobutyric acid
IVL: involucrin
MTT: 3-(4,5-dimethyl-2-yl)-2,5-diphenyltetrazolium bromide
S: sourdough
TBS: tris buffered saline
TTA: total titratable acidity
WSE: water/salt-soluble extract
